# Vaccination Diffusion and Incentive: Empirical Analysis of the US State of Michigan

**DOI:** 10.3389/fpubh.2021.740367

**Published:** 2021-09-08

**Authors:** Hwang Kim, Vithala R. Rao

**Affiliations:** ^1^Chinese University of Hong Kong (CUHK) Business School, The Chinese University of Hong Kong, Shatin, China; ^2^The Samuel Curtis Johnson Graduate School of Management, Cornell University, Ithaca, NY, United States

**Keywords:** COVID-19, vaccination rollouts, vaccination incentive, diffusion model, synthetic control method, natural experiment

## Abstract

Vaccination is the only way to reach herd immunity and help people return to normal life. However, vaccination rollouts may not be as fast as expected in some regions due to individuals' vaccination hesitation. For this reason, in Detroit, Michigan, the city government has offered a $50 prepaid card to people who entice city residents to visit vaccination sites. This study examined vaccination rates in the US using Detroit, Michigan, as the setting. It sought to address two issues. First, we analyzed the vaccination diffusion process to predict whether any region would reach a vaccination completion level that ensures herd immunity. Second, we examined a natural experiment involving a vaccination incentive scheme in Detroit and discovered its causal inference. We collected weekly vaccination data and demographic Census data from the state of Michigan and employed the Bass model to study vaccination diffusion. Also, we used a synthetic control method to evaluate the causal inference of a vaccination incentive scheme utilized in Detroit. The results showed that many Michigan counties—as well as the city of Detroit—would not reach herd immunity given the progress of vaccination efforts. Also, we found that Detroit's incentive scheme indeed increased the weekly vaccination rate by 44.19% for the first dose (from 0.86 to 1.25%) but was ineffective in augmenting the rate of the second dose. The implications are valuable for policy makers to implement vaccination incentive schemes to boost vaccination rates in geographical areas where such rates remain inadequate for achieving herd immunity.

## Introduction

The length of the COVID-19 pandemic has surpassed 1 year and continues unabated in numerous geographical locales. Although governments worldwide have implemented an array of extensive interventions to combat the COVID-19 pandemic—such as contact tracing, social distancing, border closings, and city lockdowns—the pandemic remains inadequately controlled in many areas. Among various interventions, mass vaccination seemingly is the only solution to vitiate the Covid-19 pandemic and enable people to return to normal life.

Some countries have begun expedited vaccination rollouts and achieved high vaccination rates. For example, Israel conduced a centralized vaccination program and reached the world's initial herd immunity. Also, the US, the UK, Chile, and Saudi Arabia have engaged in similarly successful vaccination efforts. Such endeavors affords countries ability to move on to a new phase of the pandemic.

As this new phase unfolds, of particular importance is whether herd immunity will be achieved through mass vaccination programs. However, there is an obstacle to vaccination rollouts: some individuals are hesitant toward—and even skepticism about—vaccination. According to recent work that investigated people's intention to receive a vaccination, a considerable proportion are averse to get vaccinated (1, 2). As such, some places in the US manifest particularly low vaccination rates (e.g., Mississippi, Alabama, Louisiana).

The foregoing phenomenon led to the current empiricism. In particular, this study investigated one incentive scheme to actuate people to get vaccinated in the US. We applied the Bass, Gompertz and Logistic diffusion model to vaccination data in the state of Michigan and predicted vaccination diffusion and likelihoods of achieving herd immunity. We then employed a synthetic control method to explore the causal effect of a vaccination incentive program implemented in the city of Detroit.

Our results revealed that the incentive scheme in Detroit increased the weekly vaccination rate by 44.19% for the first dose (from 0.86 to 1.25%) but was ineffective for the second dose. Indeed, despite the putative benefit of the vaccination incentive scheme, the Bass model predicted that Detroit would not reach a vaccination level high enough to achieve herd immunity, yet ~24.1% of counties in Michigan would likely engender a vaccination rate exceeding 60%. Given this finding, we suggest that offering incentives at an early stage and for the second doses, as well as education, and targeting certain age groups would be salutary. This study offers valuable and timely important implications for policy makers to augment vaccination rates whether vaccination rollouts are currently underway or will begin imminently.

## Data and Methodology

### Background: Good Neighbor Program in Michigan State

The US has been one of the countries that the COVID-19 pandemic has markedly adversely affected. Consequently, it was impelled—in general—to initiate expeditious nationwide vaccination rollouts and was one of very few countries where vaccines were developed. Epidemiologists have averred that the key to mitigate effectively this pandemic is to consequentially increase vaccination rates and achieve herd immunity quickly.

To this end, various incentive schemes have been offered in the US. For example, in the education sector, Wayne State University in Detroit, Michigan, provides students with a $10 credit on their accounts for receiving a vaccination (3). In the private sector, Uber and Lyft are offering free rides to and from vaccination sites between May 24, 2021, and July 3, 2021 (4). Many companies especially in the service sector—such as American Airlines, Walmart and Lidl—are offering incentives (e.g., paid vacation, cash) to their workers who get vaccinated (5).

As such, the state of Michigan planned to reopen the state and cities (e.g., relax all indoor capacity limits including those of social gatherings) if the vaccination rate hits 65%. However, as of May 4, 2021, the state of Michigan ranked 24^th^ out of the 50 US states based on the vaccination rate of those aged 16 or above. Furthermore, the rate of vaccination in Michigan had fallen to 41% after it hit a peak in April-May 2021 although the vaccination supply was not limited (6). Among all the regions in Michigan, the largest city—Detroit—has continued to display a particularly low vaccination rate. To augment this rate, Detroit's city government initiated an incentive program named “Good Neighbor” on May 3. (7)^1^

Specifically, Detroit residents need to pre-register as a Good Neighbor and make first-dose appointments for neighbors whom they then drive to their scheduled appointments. For such Detroit residents who drive their neighbors to get vaccinated for the first dose, the Detroit city is offering a $50 debit card per shot. The maximum incentive is three residents per car and per appointment trip, but it becomes unlimited if the Detroit resident finds other neighbors to take to be vaccinated. However, this program is not available to anyone under the age of 18.

This program applies for any first doses of Pfizer and Moderna or a single dose of Johnson & Johnson but is not applicable for the second doses of Pfizer and Moderna. As the US Center for Disease Control and Prevention (CDC) does not officially recommend a particular type of vaccine, the residents of Michigan are allowed to choose a clinic in terms of their preferred vaccine type. Detroit's city government received a $3.4 million state grant that will cover 68,000 Detroit residents. Despite the program's intent, an empirical question remains: Will it likely help Detroit achieve herd immunity?

### Data

**Figure 1:**
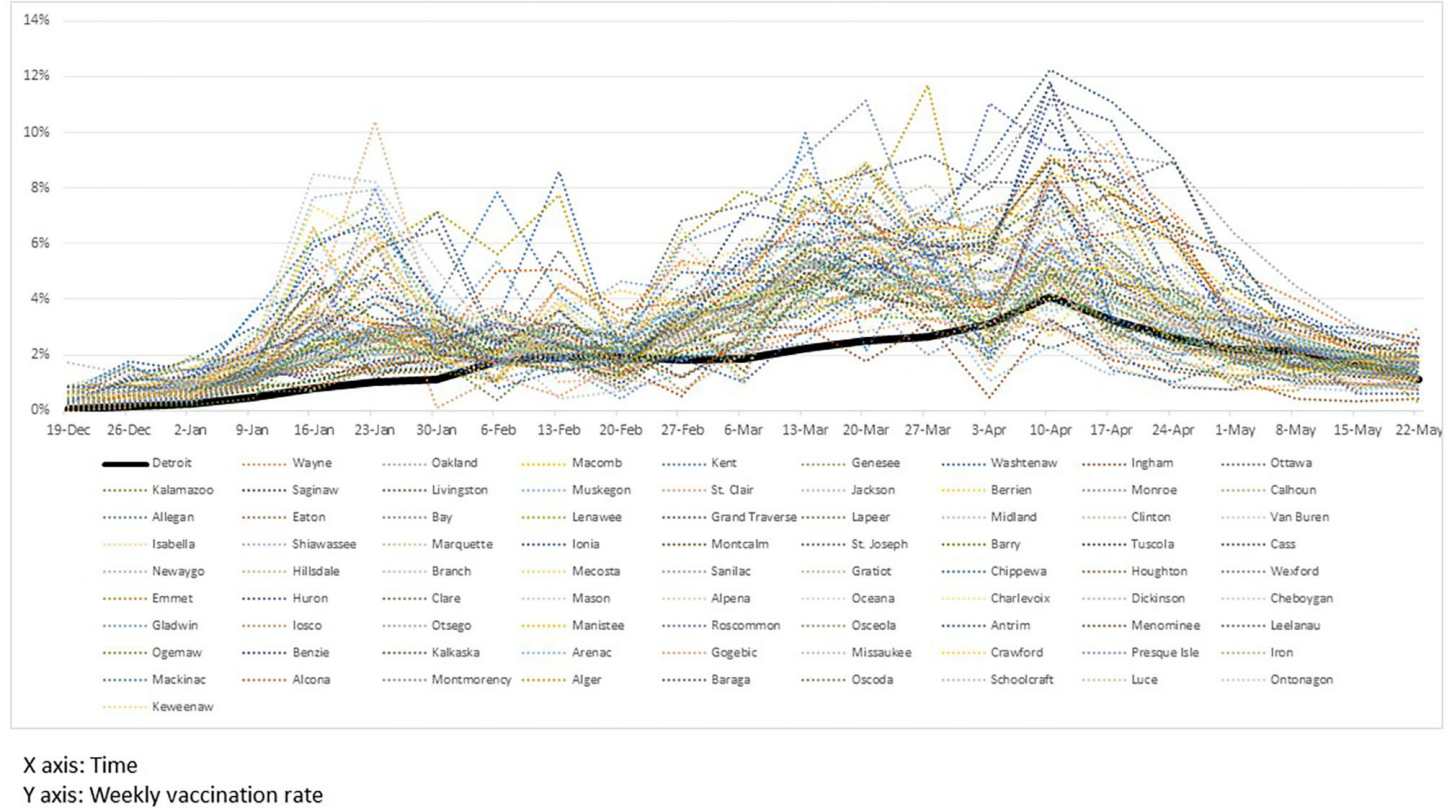
Weekly vaccination rates of counties in Michigan.

We collected vaccination data from a website, COVID-19 Vaccine Dashboard (https://www.michigan.gov/coronavirus), that provides vaccination data by county on a weekly basis in Michigan. In addition, we obtained specific demographic data in Michigan counties from Cubit (https://www.michigan-demographics.com/). Shown in Figure 1 are each county's weekly vaccination rate for 29 weeks from December 19, 2020, to July 3, 2021.

### Vaccination Diffusion

#### Bass Diffusion Model

We analyzed the vaccination diffusion process using the Bass model (8). The advantage of the Bass diffusion model is that it affords the researcher opportunity to identify what factor drives the diffusion process and estimate the potential (market) size before the process has been completed.

(1) $$\begin{array}{l} \frac{f\left(t\right)}{1-F\left(t\right)}=p+qF\left(t\right) \end{array}$$

The Bass model assumes $\frac{f\left(t\right)}{1-F\left(t\right)}$ (i.e., the portion of the potential market that adopts at time t given that they have not yet adopted) is driven by two parameters, *p* and *q*, where *f*(*t*) indicates the portion of the population that has adopted a given product at time t and *F*(*t*) reflects the portion of the population that has adopted it by time t. In the context of management and economics, parameter *p* captures the innovation effect and parameter *q* depicts the imitation effect. Specifically, there is some portion of people (innovators) who decide to adopt a product in the early stage. Following the lead of those innovators, there are individuals who adopt the product later (imitators). Also, the model assumes that everyone should adopt a product only once.

By replacing *F*(*t*) with $\frac{A\left(t-1\right)}{M}$ and *f*(*t*) with $\frac{a\left(t\right)}{M}$, where *M* indicates the potential market (e.g., the ultimate number of adopters), *a*(*t*) indicates the number of adopters at time t, and *A*(*t* − 1) indicates the number of people who have already adopted before time t, we rewrite Equation (1) as follows:

(2) $$\begin{array}{l} a\left(t\right)=\left[p+q\frac{A\left(t-1\right)}{M}\right]\left[M-A\left(t-1\right)\right] \end{array}$$

This diffusion process can be applied to our vaccination context. According to Bass diffusion, there is a type of person who decides to get vaccinated early (like innovators). S/he may be willing to take a risk (i.e., uncertainty about the vaccine's efficacy or side effects) or is eager to be immune early (owing to his/her work or age). Conversely, another type of person (like imitators) may be more cautious and thus wait to get vaccinated until after observing those who were vaccinated early. Given our vaccination context, the Bass model in (2) can be rephrased as follows in Equation (3):

(3) $$\begin{array}{l} Doses_{t}^{1}=\left[P+\frac{q}{M}{Cum\text{\_}Doses}_{it-1}^{1}\right]\left[M-{Cum\text{\_}Doses}_{it-1}^{1}\right] \end{array}$$

where,

*M*: a parameter to capture potential population who will get vaccinated eventually.
*p*: a parameter to capture innovation effect.
*q*: a parameter to capture imitation effect.
$Doses_{t}^{1}$: the number of people who receive the first dose at t, and
${Cum\text{\_}Doses}_{t-1}^{1}$: the number of people who already received the first dose until t-1
(${Cum\text{\_}Doses}_{t-1}^{1}=Doses_{1}^{1}+\ldots +Doses_{t-\;1}^{1}$).

Bass model has three parameters to be estimated; *p* (innovation effect), *q* (imitation effect) and *M* (market potential). In the context of vaccination, *M* represents the potential adoption of vaccine, which enables us to predict whether a county/city will reach herd immunity (60~70% according to WHO^2^).

#### Gompertz Diffusion Model

Gompertz model (9) is a proportional hazard model which is widely used to model diffusion processes in various domains such as Biology (10) and Epidemiology (11). Its cumulative distribution function is:

$$\begin{array}{l} {Cum\text{\_}Doses}_{t}^{1}=M\cdot exp\left(-exp\left(\frac{\mu e}{M}\left(\lambda -t\right)+1\right)\right) \end{array}$$

where *M*, λ and μ are parameters to be estimated.

#### Logistic Diffusion Model

Logistic diffusion model is a hazard model in the discrete time horizon. The logistic growth model is formulated as follows.

$$\begin{array}{l} {Cum\text{\_}Doses}_{t}^{1}=\frac{M}{1+\exp\left(\frac{4\mu }{M}\left(\lambda -t\right)+2\right)} \end{array}$$

where *M*, λ and μ are parameters to be estimated.

Note that Gompertz model and Logistic diffusion model above are the reparametrized versions. For a more discussion of such models, readers can refer to (12).

### Causality Model via Use of the Synthetic Control Method

Detroit's city-wide vaccination financial incentive program is the only one in Michigan. Therefore, it allows us to set the city of Detroit as the treatment group. As such, the other 81 counties comprise the control group.^3^

Michigan releases weekly COVID-19 vaccination data for the state's 83 counties by gender and age groups: (male, female) X (age [16~19], [20~29], [30~39], [40~49], [50~64], [65~74], [75+]), for a total of 14 groups. However, the US Census reports the population size of age groups somewhat differently—[10~19], [20~29], [30~39], [40~49], [50~59], [50~69], and [70+]. Accordingly, we were unable to match the age groups [50~64], [65~74], and [75+] in the Michigan vaccine dose data with the US Census data. To address this issue, we combined three age groups [50~64], [65~74], and [75+] into one [50+]^4^. As the Good Neighbor program reimbursement only applies to people 18+, we excluded the population group of age [16~19] for our causality analysis. Finally, we identified eight demographic groups in each county/city and their weekly vaccination rates for the first and second doses.

As mentioned earlier, the Good Neighbor program only allows the $50 incentive for people who help friends or neighbors schedule their first doses of Pfizer and Moderna and Johnson & Johnson doses^5^. We thus define a vaccination rate of first doses and Johnson & Johnson doses of demographic group g at county i at week t, ${Vaccination\text{\_}Rate}_{igt}^{1}$, implying how many people received the first dose in the population of group g in county i among those who have not received the first dose until week t-1. To ensure normality, we log-transform the variable. In this sense, this variable implies the average weekly vaccination rate:

(4) $$\begin{array}{l} {Vaccination\text{\_}Rate}_{igt}^{1}=ln\left(1+\frac{Doses_{igt}^{1}}{N_{ig}-{Cum\text{\_}Doses}_{igt-1}^{1}}\times 100\right) \end{array}$$

where,

$Doses_{igt}^{1}$: The number of first doses of group g in county i at week t
*N*_*ig*_: Population of group g in county i, and
${Cum\text{\_}Doses}_{igt-1}^{1}$: The total number of first doses of group g in county i until week t-1

To investigate causal inference using these foregoing data, an option was to aggregate these eight groups into one county-level data set and analyze the causal effect of the vaccination incentive scheme at the county level. However, doing so would have led to only nine observations (9 weeks) for the city of Detroit under the treatment (vaccination incentive) period, possibly leading to an over-fitting problem.

Thus, to avoid any over-fitting problem and make full use of the data, we analyzed these data by demographic group levels. Note that the empirical setting in this study is that there are only a few treatment groups (eight demographic groups in Detroit) and many control groups (81 × 8 demographic groups in 81 Michigan counties). For this reason, we employed a synthetic control method developed for block-level Census demographic data.

For example, for the first doses, the synthetic control method is described as follows:

$$\begin{array}{l} {Vaccination\text{\_}Rate}_{igt}^{1}={Vaccination\text{\_}Rate}_{igt}^{1}\left(0\right)+\alpha_{igt}^{1}D_{gt} \end{array}$$

${Vaccination\text{\_}Rate}_{igt}^{1}\left(0\right)$ is a vaccination rate of first doses in demographic group *g* at time t in absence of treatment, and *D*_*gt*_ is a treatment indicator (i.e., vaccination incentive in Detroit city). Thus, the key is to estimate $\alpha_{igt}^{1}$ which captures the causality effect for the treatment. Note that ${Vaccination\text{\_}Rate}_{igt}^{1}\left(0\right)$ is not observed for the treatment groups for post-intervention periods (i.e., synthetic control group). Thus, the synthetic control groups were constructed by matching treatment groups (i.e., Detroit City) and control groups (i.e., 81 Counties in Michigan) for 20 weeks of pre-intervention time periods, covariates (age and gender) and intercept. For more detail of the block-level synthetic control method, refer to (13).

The overall causal effect is estimated by averaging $\alpha_{igt}^{1}$ across eight demographic groups by gender and age. We tested this model for first doses and Johnson & Johnson doses using demographic groups in Detroit and in 81 Michigan counties for 29 weeks: (i) pre-intervention time periods (20 weeks): December 19, 2020 (the first week of the vaccination rollout in Michigan) to May 2 2021, and (ii) post-intervention time periods (9 weeks): May 3 (the first week of the Good Neighbor program in Detroit) to July 3, 2021.

Note that some of the first dose recipients due to the Good Neighbor program may take their second doses later. Thus, we apply the same method to the second doses. Similarly to Equation (4), for the second doses, we define a dependent variable as a vaccination rate of second doses of group g in county i at week t, ${Vaccination\text{\_}Rate}_{igt}^{2}$ in Equation (5), implying how many people received the second dose among people who had received the first dose of Pfizer or Modena at least 4 weeks ago^6^ and have not received the second dose until week t-1. Accordingly, we apply different post-intervention time periods (5 weeks): May 31 (4 weeks after the Good Neighbor program was launched in Detroit) to July 3, 2021.

(5) $$\begin{array}{l} \;{Vaccination\text{\_}Rate}_{igt}^{2}\\ =ln\left(1+\frac{Doses_{igt}^{2}}{{Cum\text{\_}Doses}_{igt-4}^{1}-{Cum\text{\_}Doses}_{igt-1}^{2}}\times 100\right) \end{array}$$

where,

$Doses_{igt}^{2}$: The number of second doses of group g in county i at week t, and
${Cum\text{\_}Doses}_{igt-1}^{2}$: The total number of second doses of group g in county i until week t-1.

## Results

### Vaccination Diffusion Process

We estimate Bass, Gompertz and Logistic diffusion models as described in section **Vaccination Diffusion**. Using *Non-linear Least Squares* package in R^7^. We first compare the model performance of the three models. To do so, we estimate the three models in 81 counties and Detroit in Michigan and compute their MAEs (mean square error). The result shows that Bass model considerably outperforms Gompertz and Logistic diffusion models for all counties. We report the full result in Supplementary Material 1.

**Figure 2:**
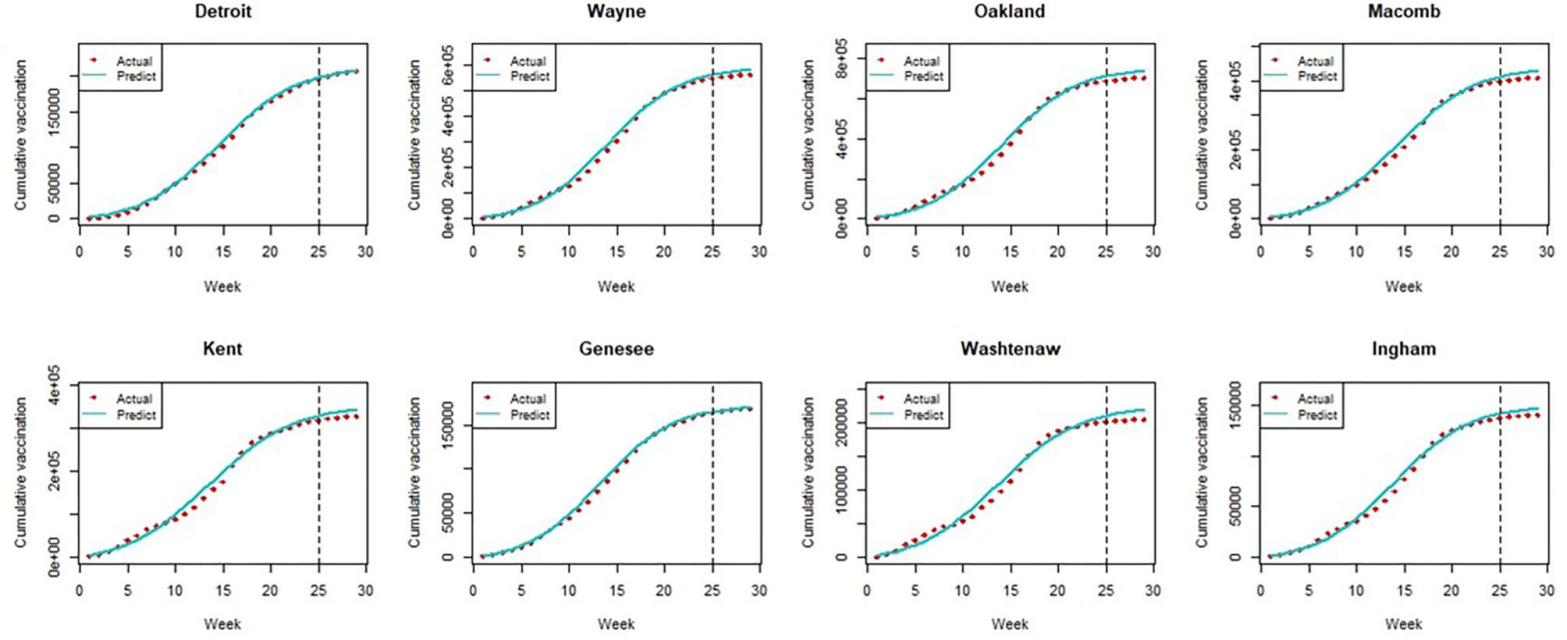
The model fit and prediction of bass model.

To access the prediction power of the Bass model, we divided the data into two parts. We estimated the Bass model using the first 25 weeks (from Dec 19, 2019, to June 5, 2020) and then predicted vaccination for the last 4 weeks (from June 12 to July 3, 2020) using the estimated parameters for 81 counties and the city of Detroit. Figure 2 shows the model fits and predictions for the seven most populated counties and Detroit. The figures reveal that predictions for those 4 weeks are very close to the actual vaccination data. For example, the predictions of Detroit and Genesee County are quite accurate for the prediction period (from June 12 to July 3) while the Bass model slightly, but only marginally, over-predicts the vaccination rate in Oakland, Macomb, and Washtenaw Counties. The figures of model fits and predictions for all 81 counties and Detroit are depicted in Supplementary Material 2. (Recall that Lake County was omitted, leaving 81 counties and Detroit.)

**Figure 3:**
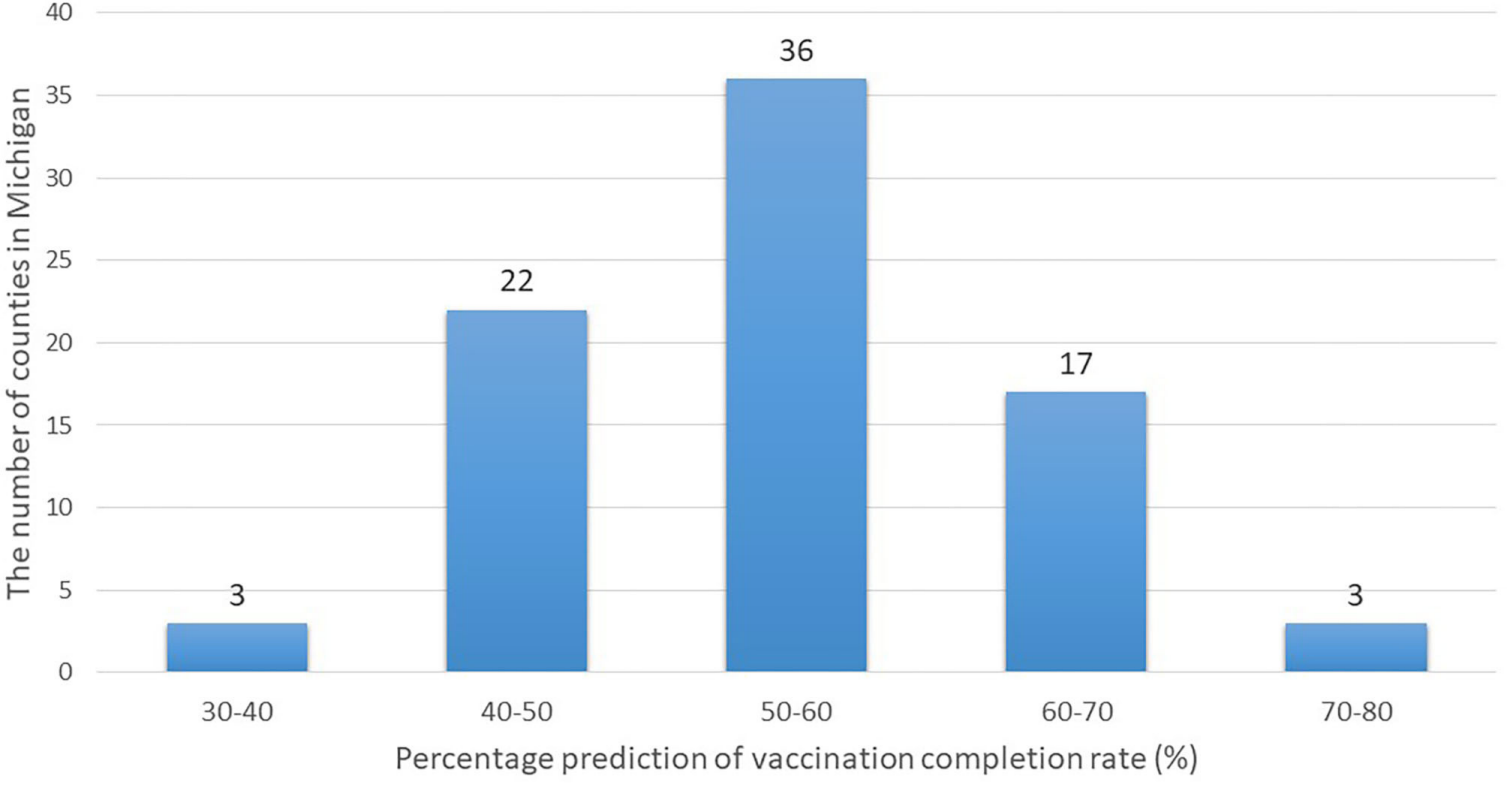
Histogram of predicted vaccination completion rates of counties in Michigan.

Next, shown in the histogram in Figure 3 are the predicted vaccination completion rates based on estimates of the Bass model for 81 counties and Detroit. Twenty counties out of 81 are expected to reach a herd immunity range eventually (at least a 60% vaccination rate). However, the vaccination completion rate is expected to achieve only a 39.9% rate in Detroit. While recent studies have predicted relatively high acceptance of vaccination in the US based on surveys [e.g., 67% in (14); 79% in (15)], vaccination rates are unlikely to be as high as projected in some regions, as shown in our analysis of the Michigan data.

The Bass model estimation depends chiefly on several factors—such as whether the diffusion spreads quickly in the early stage and the height of its peak if the data include the diffusion's peak. As shown in Figure 1, in the earlier stage of the vaccination process, most counties had passed the peak as of May 22, 2021. Then, the size of the potential population (i.e., how many people would receive the first dose eventually in our context) was driven by the value at the peak. Because the peak in Detroit was particularly lower than in counties with a similar population size, the estimate of the potential population size was also estimated to be lower.

To address this problem further, we estimated the Bass model using seven demographic groups in Detroit City, the results of which are reported in Table 1. Interestingly, we found a trend in the vaccination completion rate by age. Specifically, the more elderly group (age 50 or above) would reach a 60% completion rate, but this trend decreased for younger age groups.

**Table 1:** Bass model results for detroit.

**Group**		**Population**	**Potential total vaccinated people**	**Innovation parameter**	**Imitation parameter**	**Expected percentage of vaccinated people**
Female		276,057	117,116	0.007	0.231	42.42%
Male		244,087	97,139	0.005	0.244	39.80%
Age	16~19	57,083	7,092	0.000	0.425	12.42%
	20~29	91,284	21,932	0.001	0.291	24.03%
	30~39	83,994	25,489	0.002	0.277	30.35%
	40~49	87,068	29,368	0.001	0.295	33.73%
	50 or above	200,715	126,061	0.007	0.264	62.81%

This finding is reasonable in that elderly people are more willing to become vaccinated as they are more vulnerable to the virus, and the US government promoted the vaccination of elderly people. However, younger age groups may believe that they are invulnerable to the virus. Although young people may have low infection rates, they may be careless in their behaviors, which may affect other vulnerable groups. Thus, policymakers should develop particular strategies to target young people (e.g., vaccination incentives, social media campaigns, and promotions).

### Causal Inferences of Vaccination Incentive

We estimated the synthetic control method in section Causality Model via Use of the Synthetic Control Method using the R package, *miscrosynth*, developed by (16). First, we found a significant impact of the vaccination incentive scheme in Detroit for the first doses: a 30.4% increase compared to a synthetic control group (*p*-value = 0.000). Note that the dependent variable is a log-transformed vaccination percentage.

Specifically, the synthetic control method result reports the dependent variable in Equation (3), ${Vaccination\text{\_}Rate}_{igt}^{1}=ln\left(1+\frac{Doses_{igt}^{1}}{N_{ig}-{Cum\text{\_}Doses}_{igt-1}^{1}}\times 100\right)=$0.808 for the treatment group (i.e., Detroit) and ${Vaccination\text{\_}Rate}_{igt}^{1}=ln\left(1+\frac{Doses_{igt}^{1}}{N_{ig}-{Cum\text{\_}Doses}_{igt-1}^{1}}\times 100\right)\;=$0.620 for the synthetic control group created by 81 counties in Michigan during the post-intervention period (9weeks from May 3 to July 3).

By transforming them to the average weekly vaccination rates, $\frac{Doses_{igt}^{1}}{N_{ig}-{Cum\text{\_}Doses}_{igt-1}^{1}}$, the result shows that the average weekly vaccination rate of 8 demographic groups during the post-intervention period was $\frac{Doses_{igt}^{1}}{N_{ig}-{Cum\text{\_}Doses}_{igt-1}^{1}}=$ 1.25 and 0.86% for the treatment group and synthetic control group, respectively. This implies that the Good Neighbor incentive program would increase the weekly vaccination rates of first doses by 44.19% (from 0.86 to 1.25%).

**Figure 4:**
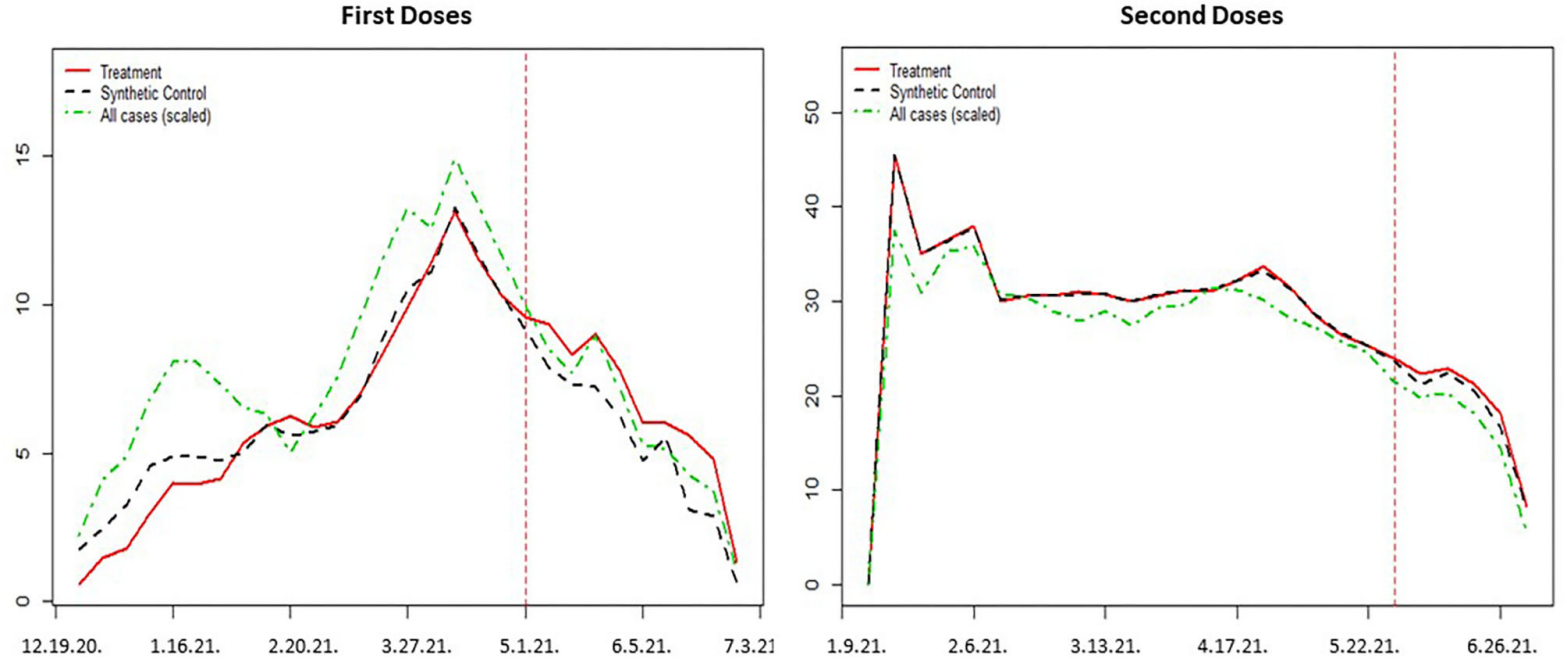
Comparison between the treatment group and synthetic control group.

Although the Great Neighbor program offers $50 prepaid cards only for first doses of Pfizer and Moderna, People who took their first doses due to the financial incentive may take their second doses later on. This can be considered as an indirect effect of the Great Neighbor incentive program. However, we found that the impact of the vaccination incentive scheme in Detroit was not statistically significant for the second doses 4 weeks after the Great Neighbor program started. In fact, we observed a 4.7% increase compared to a synthetic control group, but its *p*-value was 0.156. Portrayed in Figure 4 are the results illustrated for the first and second doses vis-à-vis the synthetic control groups.

Note that this implication may be different for the Johnson & Johnson vaccine which requires only a single dose. That is, when incentives are offered only for one dose (i.e., first dose), the vaccine which requires only a single shot, such as Johnson & Johnson, would seem to become more appropriate for the Good Neighbor program. However, because the Johnson & Johnson vaccine was not popular with people (only 6.42% received the Johnson & Johnson vaccine in our data), limiting the effect of the Good Neighbor program only to the Johnson & Johnson vaccine would underestimate its effect.

According to a report released by the US CDC (17) in March 2021, a single dose of Pfizer or Moderna vaccines is 82% effective against symptomatic COVID-19 (while two doses are 94% effective). This implies that the Good Neighbor vaccination incentive may seem somewhat effective despite the insignificant result for second doses.

However, the COVID-19 pandemic recently began to enter a new phase. The Delta variant is the predominant strain of the virus all over the world. A new and big wave of COVID-19 infections has been fueled by the Delta variant after a long decline in the number of COVID-19 cases, as the Delta variant is almost twice as contagious as previous variants^8^. Thus, the previous study on vaccine effectiveness [e.g., the CDC report released in March (17)] may be no longer valid.

Recently in August 2021, a medical study reports that the effectiveness after one dose of Pfizer vaccine was notably low for the Delta variant (36%), whereas the effectiveness of two doses of Pfizer was 88% (18)^9^. In response to the Delta variant surge, it is strongly recommended that the vaccination incentive program should be applied to the second doses.

## Discussion

Individuals' vaccine hesitancy remains a barrier in vitiating the COVID-19 pandemic and achieving herd immunity. Our effort was the inaugural empirical study to investigate the effectiveness of vaccination incentive programs as a government intervention using actual vaccination data. To determine the causal inference, our context was in the US state of Michigan, where one of its cities, Detroit, has implemented a city-wide vaccination financial incentive program for its residents. We used a synthetic control method to uncover the causal effect.

Our analysis showed that the financial incentive scheme increased the vaccination rate effectively, raising the weekly vaccination rate for the first dose from 0.86 to 1.25%. However, this scheme was seemingly unable to change the situation favorably completely. Given Detroit's low vaccination rate, the Bass model predicted that the financial incentive may not be sufficiently efficacious to afford Detroit's achievement of a level of herd immunity—as shown in section Vaccination Diffusion Process.

The finding of our study is particularly important as other states (or countries) implement similar vaccination incentive programs. For example, in North Carolina, USA, people who drive others to vaccination centers are awarded a $25 cash card after their passenger's vaccination, which is similar to the Good Neighbor program in Detroit. Note that the purpose of this incentive program is to remove any transportation barrier and to persuade other people to become vaccinated. Thus, our findings are crucial especially in the US as 45% of Americans have no access to public transportation^10^ Policymakers should consider this incentive program, especially where transportation is rather limited for vulnerable people such as the poor and elderly or where the public transit infrastructure is poor. Therefore, our analysis in the state of Michigan will provide useful implications for policymakers in other such places.

Interestingly, there are two other popular vaccination incentives implemented in the US. First, lottery-type incentive programs have been implemented in other US states. For example, the US state of Ohio began offering lottery tickets worth $1 million over 5 weeks for newly vaccinated residents on May 26, 2021. Similarly, the states of Colorado, Maryland, and Washington are conducting lottery cash drawings for newly vaccinated individuals.

The second type of incentive is to provide cash directly to vaccinated individuals, not just the people who drive others to get vaccinated. For example, the West Virginia government provides a $100 saving bond or gift card to young people between 16 and 35 who receive the vaccine. Also, US President Biden has called on states a policy at July 30, 2020, to encourage people to get vaccinated, for example paying them $100 cash. For various vaccination incentive programs in the US, refer to (19). Future studies could investigate various incentive schemes and, by comparing their effects with our findings, suggest the best incentive type given specific situations (e.g., big city, small town, rich/poor cities, public/mass transit infrastructure).

In addition, as mass vaccination rollouts have progressed, a concern for policy makers is that about 8% of the people in the US who have received their first dose may miss their second dose (20). Indeed, when applying Detroit's incentive scheme to those who take the second dose, we did not find any empirical evidence that this effort was effective for increasing the rate of second doses. As mentioned earlier, the vaccine effectiveness against the Delta variant is high only after two doses. Therefore, policy makers should consider providing incentives to people to get vaccinated with their second dose.

Detroit's incentive scheme of offering a financial benefit for being vaccinated may not be very effective because people currently may perceive less risk (21). If so, we can infer that the incentive scheme and promotion campaigns must be planned and implemented in the *early* phase of a vaccination rollout. In addition to the financial incentive effort, promotion through education (e.g., about vaccine side effects or its reduction in risk) may be useful to enhance people's acceptance of the COVID-19 vaccine (22). Also, because our Bass model predicted that younger age groups (age 16~49) would realize very low vaccination rates, an incentive scheme targeting a particularly younger age group seems necessary. For example, South Korea announced vaccination incentives—such as wearing no mask outside and traveling free without a mandatory quarantine—for individuals vaccinated on May 28, 2021. It led to a sharp increase in the trend of the search term “find vaccination sites” among young people on a major search engine, Naver.

To summarize, providing incentive schemes to increase COVID-19 vaccination rates is an important intervention policy for governments to help people return to normal life. Despite the importance of doing so, whether such vaccination incentive schemes are effective was unknown until our current undertaking. We used the Bass model to predict whether any geographical locale would reach vaccination rates requisite to achieve herd immunity. Also, our study provided empirical evidence of the potential benefit of offering an incentive scheme: such an effort engendered a significantly favorable impact on the first dose rate but not on the second dose rate. Our work thus led to crucial and timely recommendations for health policy makers in countries where the vaccination rollout is slow.

Our study is without limitation. Our context—the US state of Michigan—releases the number of vaccinated people by age and gender but not by such other factors as education or income. As such, scholars should explore this issue by incorporating these characteristics to evaluate the efficacy of vaccination incentive programs in enhanced detail.

## Data Availability Statement

Publicly available datasets were analyzed in this study. This data can be found at: https://www.michigan.gov/coronavirus https://www.michigan-demographics.com.

## Author Contributions

HK: conceptualization, writing, data analysis, and original draft preparation. VR: review and editing. Both authors contributed to the article and approved the submitted version.

## Conflict of Interest

The authors declare that the research was conducted in the absence of any commercial or financial relationships that could be construed as a potential conflict of interest.

## Publisher's Note

All claims expressed in this article are solely those of the authors and do not necessarily represent those of their affiliated organizations, or those of the publisher, the editors and the reviewers. Any product that may be evaluated in this article, or claim that may be made by its manufacturer, is not guaranteed or endorsed by the publisher.

## Footnotes

^1^The Good Neighbor program that initially started in Detroit earlier allowed adults age 55+ (who were at the time ineligible) the opportunity to access the vaccine if they brought an adult age 65+ to a vaccination center. However, we do not consider this initial program to be relevant as our focus is only to analyze the causal effect of the financial incentive ($50 prepaid card) that started on May 3rd. For more detail, refer to an article at https://detroitmi.gov/departments/detroit-health-department/programs-and-services/communicable-disease/coronavirus-covid-19/covid-19-vaccine/good-neighbor-program.

^2^https://www.who.int/emergencies/diseases/novel-coronavirus-2019/media-resources/science-in-5/episode-1.

^3^We excluded Lake County for our analysis, because there was a week when the weekly vaccination rate was abnormally 7.6 times higher than the average rate; unfortunately, we could not identify the reason.

^4^Although adults of age 65+ had access to the vaccine in Michigan two months prior to adults of age 50+, we cannot distinguish the age groups of 50-65 and 65+ in the US Census data. Due to this data limitation, we grouped them into one group.

^5^There were 311,648 doses of Johnson & Johnson (6.42%) among 4,849,774 first doses in Michigan State during the data period.

^6^The minimum amount of time between doses is 3 weeks for Pfizer and 4 weeks for Moderna.

^7^https://stat.ethz.ch/R-manual/R-devel/library/stats/html/nls.html.

^8^https://www.cdc.gov/coronavirus/2019-ncov/variants/delta-variant.html.

^9^The study also reports the results of AstraZeneca, which effectiveness is 30% for one doses and 67% for two doses for the Delta. But, the study did not investigate the effectiveness of Moderna and Johnson & Johnson vaccines.

^10^https://www.apta.com/news-publications/public-transportation-facts/.
