# Supplementary material for: Vaccination Diffusion and Incentive: Empirical Analysis of the US State of Michigan
Source: Front Public Health. 2021 Sep 8;9:740367. doi: 10.3389/fpubh.2021.740367 (PMC8457353; doi:10.3389/fpubh.2021.740367)
Supplement: Supplementary file 1 [file Data_Sheet_1.PDF]

Supplementary Material 1

Supplementary Table 1. Estimation Results for Counties in Michigan State

County in Michigan	MAE (mean square error)			Bass Model Results		
	Bass	Gompertz	Logistic	Potential total vaccinated people	population (age 16+)	Expected % of vaccinated people
Leelanau	6.11E+04	6.58E+07	5.68E+07	14371	18819	76.4%
Grand Traverse	1.89E+06	1.09E+09	8.79E+08	55726	76864	72.5%
Washtenaw	5.23E+07	1.99E+10	1.54E+10	214523	304782	70.4%
Oakland	3.17E+08	2.39E+11	1.88E+11	730168	1039050	70.3%
Emmet	8.09E+04	1.24E+08	1.05E+08	19270	27988	68.9%
Benzie	3.57E+04	3.61E+07	2.99E+07	10008	15045	66.5%
Kent	9.06E+07	5.11E+10	3.90E+10	342513	517675	66.2%
Charlevoix	6.52E+04	7.05E+07	5.96E+07	14426	22143	65.2%
Marquette	7.82E+05	4.32E+08	3.57E+08	36381	56091	64.9%
Clinton	1.03E+06	6.79E+08	5.45E+08	41323	64140	64.4%
Mason	1.09E+05	8.10E+07	6.70E+07	15444	24178	63.9%
Kalamazoo	6.24E+06	7.15E+09	5.84E+09	135841	214438	63.3%
Ottawa	1.20E+07	8.35E+09	6.62E+09	142840	227749	62.7%
Midland	7.31E+05	6.64E+08	5.53E+08	42945	68620	62.6%
Alger	2.08E+04	6.46E+06	5.85E+06	4961	7977	62.2%
Mackinac	2.42E+04	8.38E+06	7.51E+06	5879	9461	62.1%
Livingston	5.74E+06	4.15E+09	3.33E+09	96764	156945	61.7%
Ingham	1.26E+07	9.32E+09	7.40E+09	145596	238053	61.2%
Manistee	6.91E+04	5.49E+07	4.65E+07	12915	21118	61.2%
Eaton	9.76E+05	1.25E+09	1.01E+09	55053	90628	60.7%
Presque Isle	2.50E+04	1.43E+07	1.27E+07	6717	11093	60.5%
Antrim	2.17E+04	4.96E+07	4.29E+07	11832	19831	59.7%
Keweenaw	8.95E+02	3.43E+05	2.98E+05	1107	1860	59.5%
Bay	3.75E+05	9.86E+08	8.26E+08	51607	87057	59.3%
Macomb	7.62E+07	8.40E+10	6.54E+10	426449	720809	59.2%
Otsego	5.83E+04	4.75E+07	3.99E+07	11561	20303	56.9%
Muskegon	1.14E+06	2.42E+09	1.97E+09	80015	140568	56.9%
Van Buren	2.33E+05	4.87E+08	3.98E+08	34570	61115	56.6%
Huron	1.68E+05	7.83E+07	6.49E+07	14933	26438	56.5%
Iosco	2.59E+04	5.35E+07	4.64E+07	12232	21761	56.2%
Wexford	9.63E+04	8.59E+07	6.89E+07	15122	26975	56.1%
Ontonagon	1.37E+04	2.09E+06	1.87E+06	2966	5294	56.0%
Alcona	5.22E+03	8.48E+06	7.54E+06	5185	9275	55.9%

Alpena	8.32E+04	5.53E+07	4.79E+07	13535	24250	55.8%
Jackson	1.53E+06	2.00E+09	1.60E+09	72553	130555	55.6%
Oceana	3.89E+04	5.39E+07	4.43E+07	11954	21521	55.5%
Roscommon	2.34E+04	5.13E+07	4.39E+07	11611	20915	55.5%
Houghton	3.05E+05	1.02E+08	7.80E+07	16282	29378	55.4%
Crawford	6.45E+03	1.44E+07	1.21E+07	6517	11827	55.1%
Saginaw	1.52E+06	2.80E+09	2.34E+09	86498	157338	55.0%
Allegan	5.09E+05	1.08E+09	8.78E+08	51339	93429	55.0%
Cheboygan	5.17E+04	4.86E+07	4.23E+07	12064	22064	54.7%
Berrien	1.80E+06	1.82E+09	1.49E+09	68670	126248	54.4%
Schoolcraft	1.23E+04	2.99E+06	2.71E+06	3754	6916	54.3%
Montmorency	9.48E+03	6.32E+06	5.55E+06	4428	8183	54.1%
Chippewa	1.12E+05	6.77E+07	6.15E+07	17156	31856	53.9%
Shiawassee	1.87E+05	3.92E+08	3.18E+08	30344	56517	53.7%
Arenac	3.32E+03	1.60E+07	1.39E+07	6728	12873	52.3%
Barry	1.08E+05	2.68E+08	2.18E+08	25823	49706	52.0%
Missaukee	1.01E+04	1.47E+07	1.23E+07	6331	12190	51.9%
Genesee	1.59E+06	1.23E+10	1.01E+10	172296	332528	51.8%
Kalkaska	2.16E+04	1.86E+07	1.54E+07	7593	14668	51.8%
Dickinson	9.07E+04	3.77E+07	3.16E+07	11030	21357	51.6%
St. Clair	2.12E+06	2.05E+09	1.64E+09	68462	132775	51.6%
Gladwin	3.57E+04	4.19E+07	3.55E+07	10932	21308	51.3%
Calhoun	4.58E+05	1.20E+09	9.63E+08	55502	108954	50.9%
Iron	2.76E+04	6.16E+06	5.51E+06	4882	9681	50.4%
Lenawee	6.98E+05	6.45E+08	5.12E+08	40505	81431	49.7%
Ionia	1.86E+05	2.69E+08	2.23E+08	25982	52448	49.5%
Lapeer	2.43E+05	5.60E+08	4.58E+08	35593	73304	48.6%
Tuscola	2.77E+05	1.74E+08	1.41E+08	21278	44045	48.3%
Clare	2.64E+04	5.23E+07	4.48E+07	12255	25571	47.9%
Gratiot	6.30E+04	9.39E+07	7.65E+07	16172	33966	47.6%
Newaygo	8.57E+04	1.40E+08	1.11E+08	18762	39637	47.3%
Isabella	1.78E+05	2.92E+08	2.38E+08	27376	58258	47.0%
Gogebic	3.45E+04	1.09E+07	9.30E+06	6086	13085	46.5%
Ogemaw	1.07E+04	2.41E+07	2.02E+07	8174	17786	46.0%
Monroe	2.09E+06	1.50E+09	1.13E+09	56976	123979	46.0%
Sanilac	2.07E+05	8.50E+07	7.08E+07	15550	34113	45.6%
Mecosta	8.16E+04	9.81E+07	7.93E+07	16122	35828	45.0%
Oscoda	2.38E+03	3.70E+06	3.17E+06	3134	6968	45.0%
Montcalm	1.44E+05	2.27E+08	1.82E+08	23311	51891	44.9%
Osceola	3.50E+04	2.90E+07	2.32E+07	8555	19084	44.8%
St. Joseph	1.19E+05	1.84E+08	1.54E+08	21729	48711	44.6%

Baraga	1.35E+04	2.33E+06	2.06E+06	3196	7226	44.2%
Luce	1.14E+04	1.21E+06	1.11E+06	2406	5453	44.1%
Branch	6.45E+04	8.23E+07	6.99E+07	15386	35390	43.5%
Wayne	9.53E+07	1.50E+11	1.19E+11	579462	1415964	40.9%
Hillsdale	6.81E+04	7.76E+07	6.45E+07	15112	37471	40.3%
Detroit	4.35E+06	2.26E+10	1.71E+10	214475	537535	39.9%
Cass	5.10E+04	9.70E+07	8.39E+07	16143	42878	37.6%
Menominee	1.73E+04	1.17E+07	1.05E+07	6107	19647	31.1%
