# Supplementary figures and images for: Vaccination Diffusion and Incentive: Empirical Analysis of the US State of Michigan
Source: Front Public Health. 2021 Sep 8;9:740367. doi: 10.3389/fpubh.2021.740367 (PMC8457353; doi:10.3389/fpubh.2021.740367)

## Supplementary Material 2

### Supplementary Figure. Model fit and prediction of Bass model

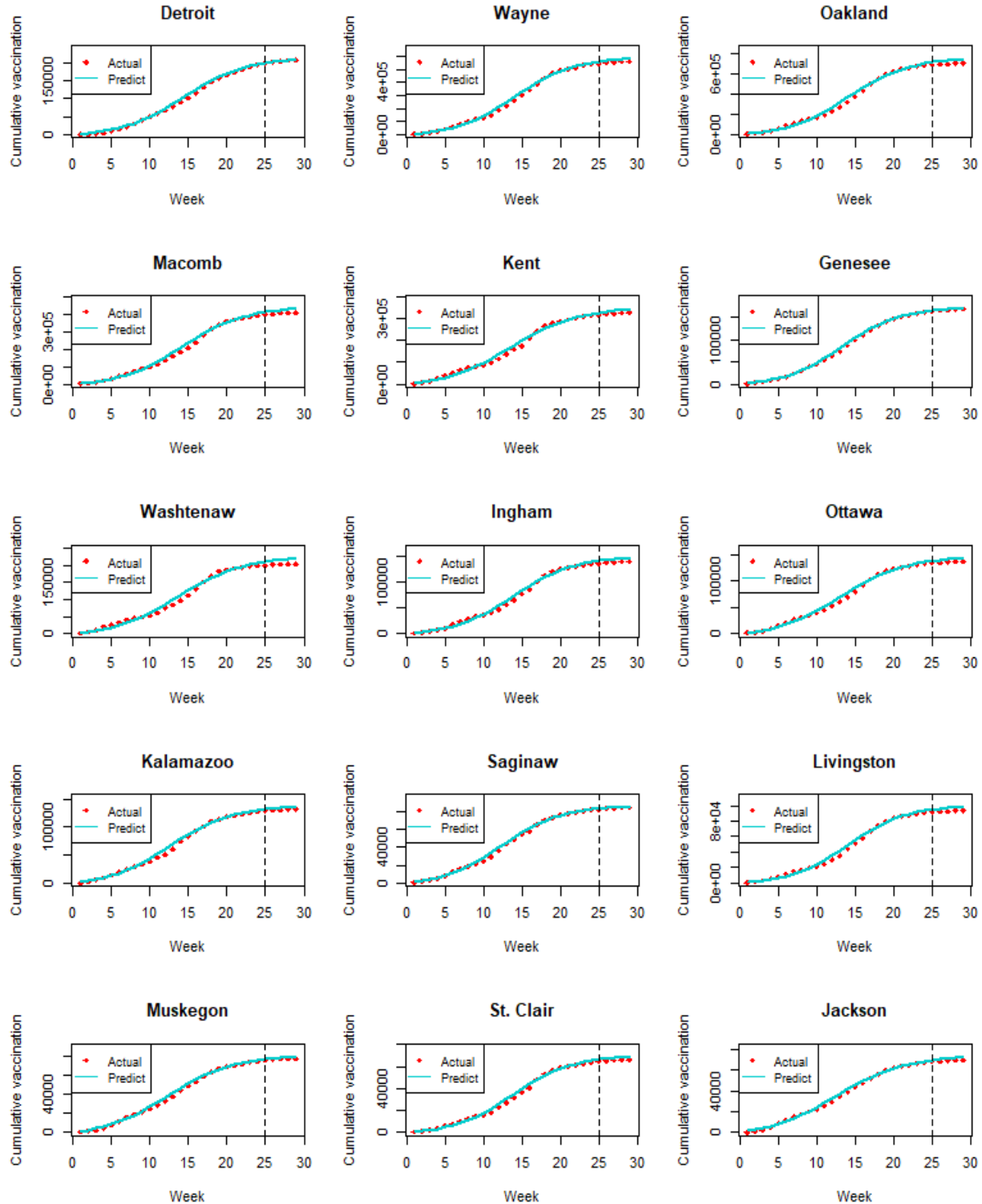

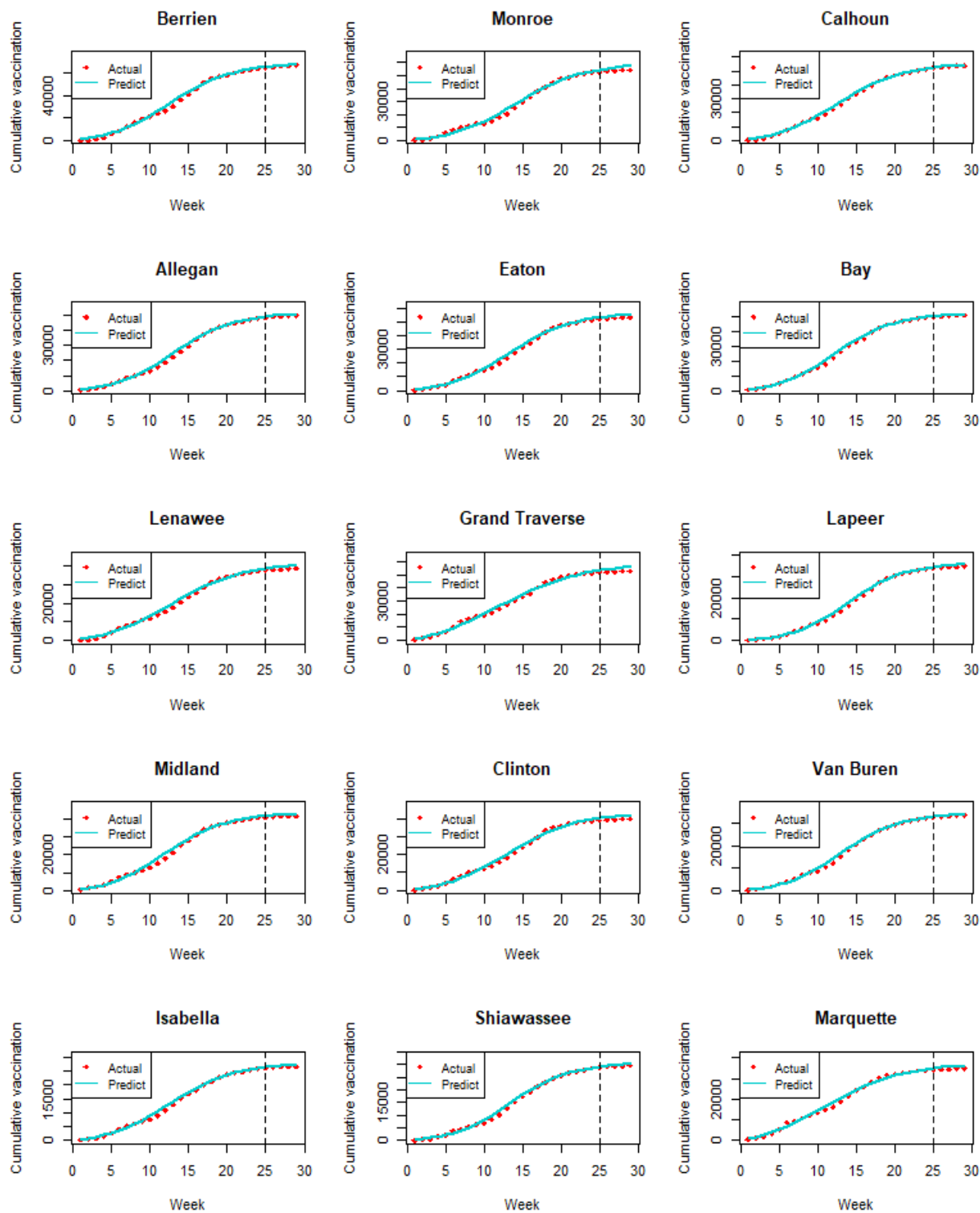

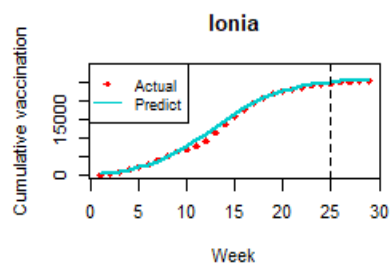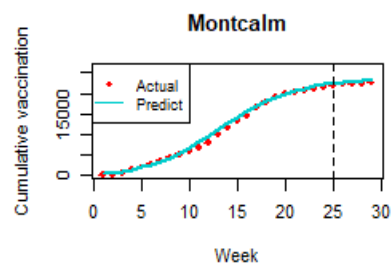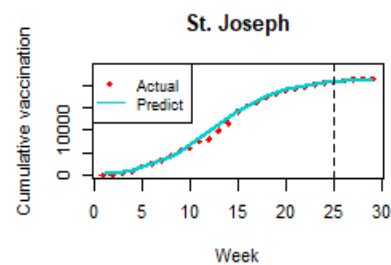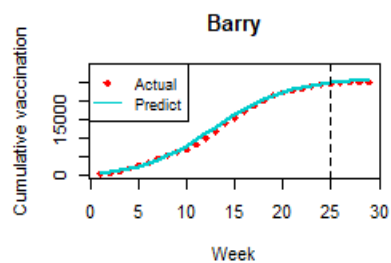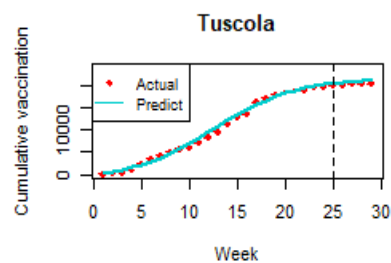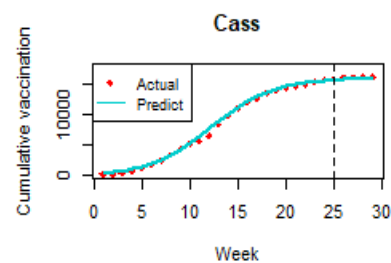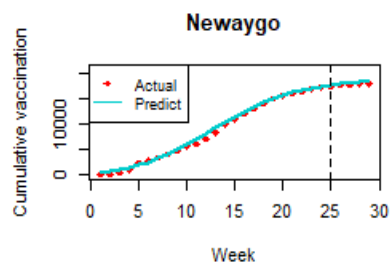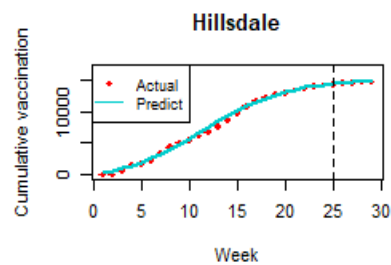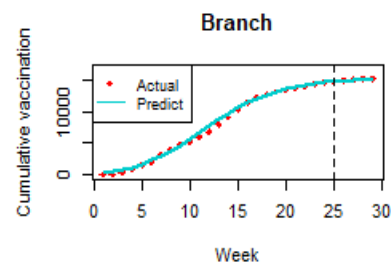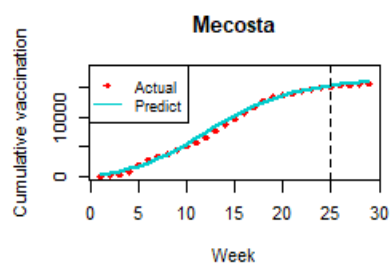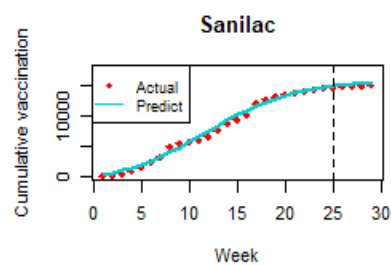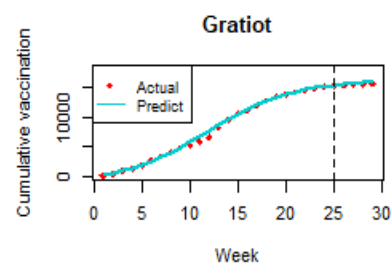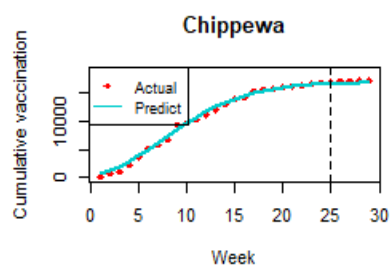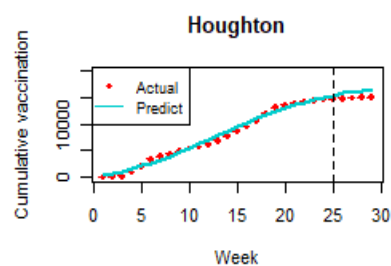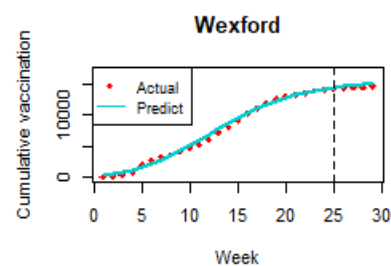

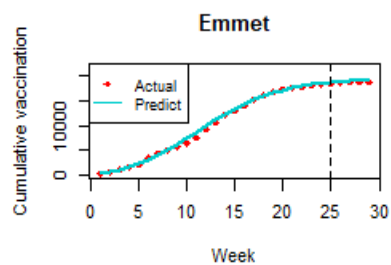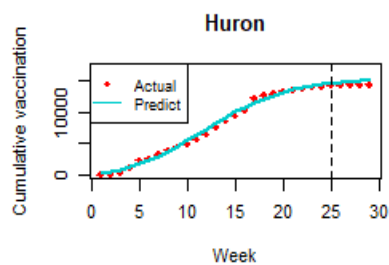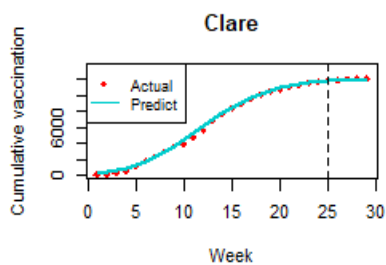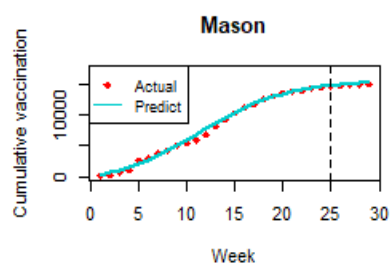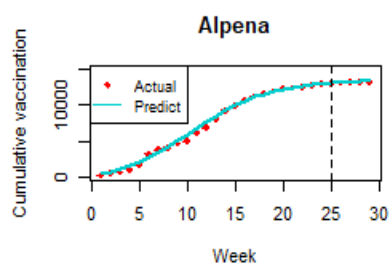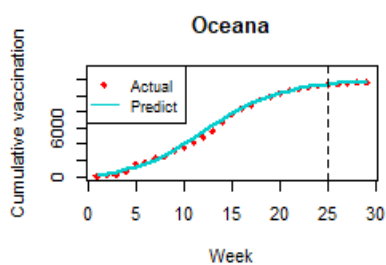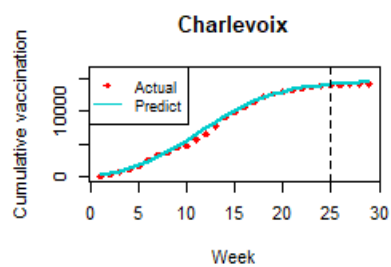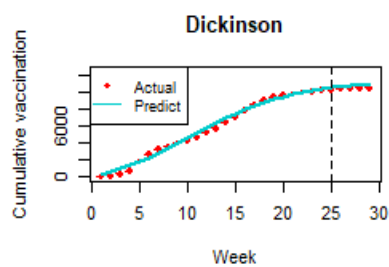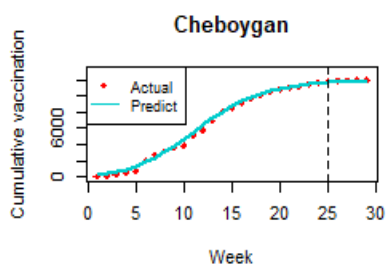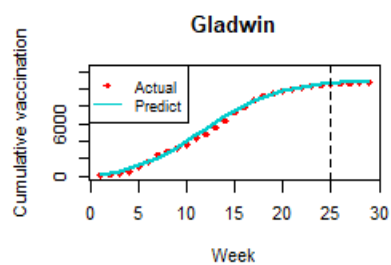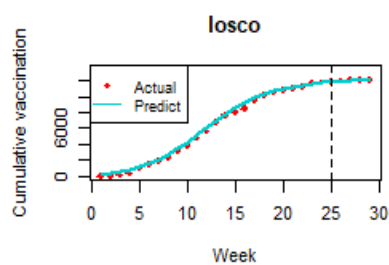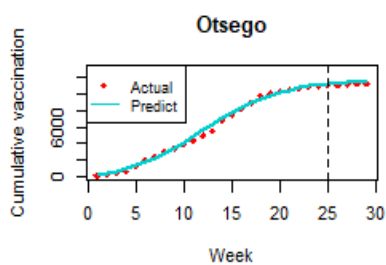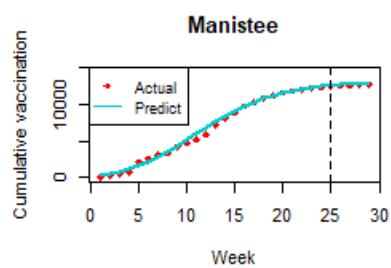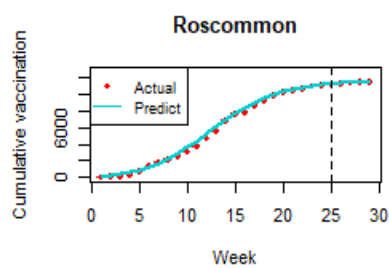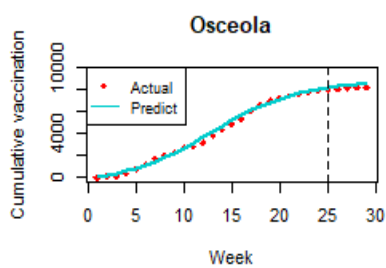

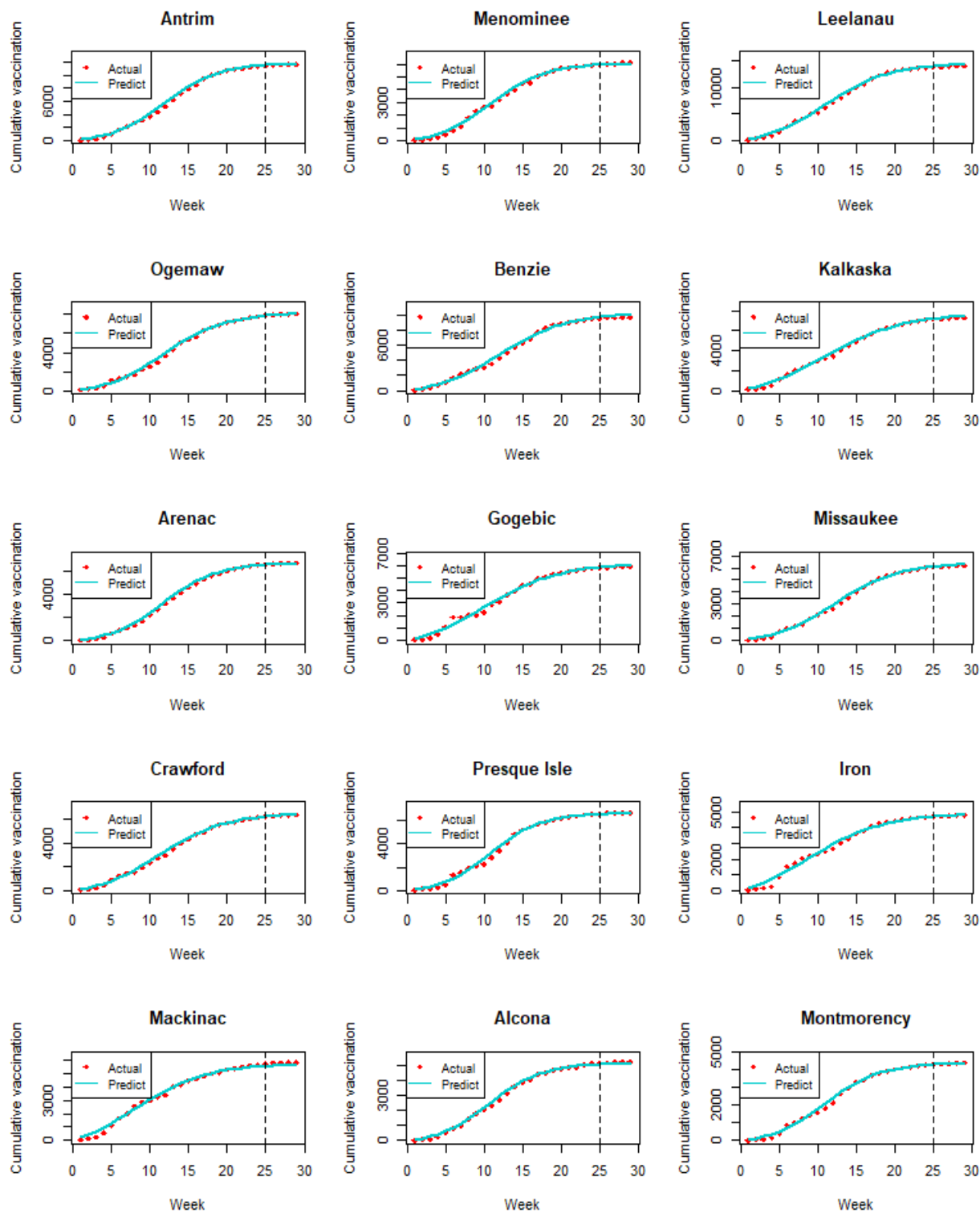

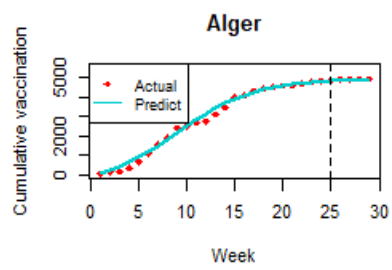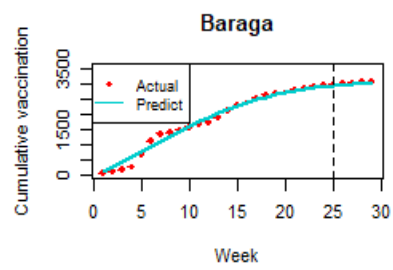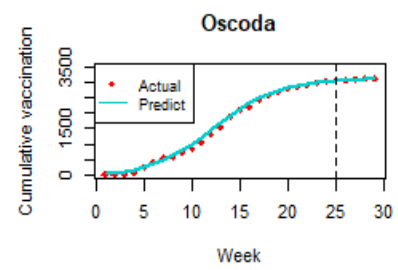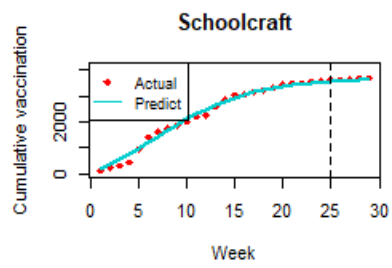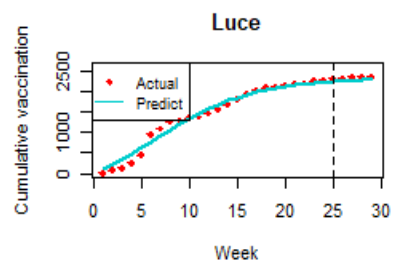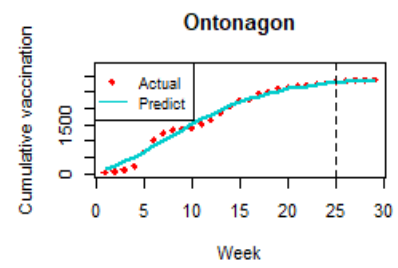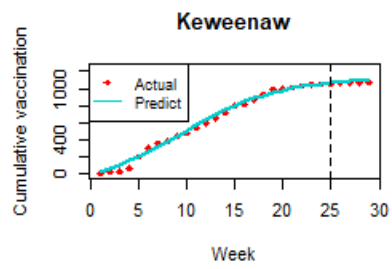

Supplement: Supplementary file 2 [file Data_Sheet_2.PDF]
